# Complete Genome Sequence of *Streptococcus* sp. Strain NPS 308

**DOI:** 10.1128/genomeA.01349-16

**Published:** 2016-12-22

**Authors:** Yoshio Kondo, Yoshitoshi Ogura, Keiko Sato, Keigo Imamura, Tomonori Hoshino, Miyuki Nishiguchi, Tomoyuki Hasuwa, Hiroyuki Moriuchi, Tetsuya Hayashi, Taku Fujiwara

**Affiliations:** a Department of Pediatric Dentistry, Nagasaki University Graduate School of Biochemical Sciences, Nagasaki, Japan; b Department of Bacteriology, Faculty of Medical Sciences, Kyushu University, Fukuoka, Japan; c Division of Microbiology and Oral Infection, Department of Molecular Microbiology and Immunology, Nagasaki University Graduate School of Biochemical Sciences, Nagasaki, Japan; d Department of Pediatrics, Nagasaki University Graduate School of Biochemical Sciences, Nagasaki, Japan

## Abstract

*Streptococcus* sp. strain NPS 308, isolated from an 8-year-old girl diagnosed with infective endocarditis, likely presents a novel species of *Streptococcus*. Here, we present a complete genome sequence of this species, which will contribute to better understanding of the pathogenesis of infective endocarditis.

## GENOME ANNOUNCEMENT

*Streptococcus* species are Gram-positive facultatively anaerobic cocci that are widely present in the oral cavity, occupying an established part of commensal flora (1). Infective endocarditis (IE) is characterized by formation of the vegetation that is a mass of platelets, fibrin, microcolonies of microorganisms, and inflammatory cells on the surfaces of the endocardium, particularly heart valves, and oral viridans streptococci are one of the major pathogens of IE (2).

The sequenced strain, *Streptococcus* sp. NPS 308, was isolated from blood drawn from an 8-year-old girl who had a history of ventricular septal defect since the neonatal period and was hospitalized for fever of unknown origin in Nagasaki University Hospital, Japan. The echocardiography showed the vegetation near the apex of the tricuspid valve, and she was diagnosed with IE in accordance with Duke diagnostic criteria. She had undergone no invasive dental treatment before the onset of IE. However, since oral streptococci were detected in blood culture, an intraoral route of infection was suspected in this case.

The physiological and biochemical features indicated the strain as *Streptococcus* sp. Moreover, phylogenetic analysis based on 16S rRNA gene sequence placed the strain among streptococci; however, classification of the strain to the species level was still inconclusive.

Bacterial cells of *Streptococcus* sp. NPS 308 were inoculated into brain heart infusion broth and incubated under anaerobic conditions. Genomic DNA was extracted using MasterPure Gram-positive DNA purification kit (Epicentre), according to the manufacturer’s introduction. Single-molecule real-time sequencing reads were generated using the PacBio RSII sequencer. Whole-genome sequencing produced a total of 162,753 reads with a mean length of 6,974 bp. Subsequent *de novo* assembly utilizing the HGAP3 protocol yielded a single scaffold with 314-fold average reference coverage. To correct sequence errors, Illumina short-read sequencing was performed using the illumina GAIIx (50-bp single-end run). By mapping the obtained 5,829,284 reads to the initial genome assembly using BWA (3) and SAMtools (4), a total of eight errors (one mismatch and seven insertions/deletions) were corrected. The final assembly contains 1,924,728 bp, with a G+C content of 41.09%. The genome sequence was annotated by Prokka version 1.11 (5) and identified 1,828 coding sequences, 62 tRNAs, four rRNA clusters, and one repeat region.

The average nucleotide identity (ANI) (6, 7) demonstrated that *Streptococcus* sp. NPS 308 differed slightly from mitis phylogenetic group of *Streptococcus*, showing ANI values of <95% compared to the genomes of the closest species, such as *Streptococcus oralis* ATCC 35037 (90.9%) and Uo5 (90.8%) (8). The alignment between *S. oralis* Uo5 and NPS 308 showed that there was no significant genome rearrangement. PHAST (9) program identified two prophage regions within *Streptococcus* sp. NPS 308.

The complete genome sequence of *Streptococcus* sp. NPS 308 will help in establishing a new species of *Streptococcus* and will provide insight into the pathogenicity of this species and host interaction.

### Accession number(s).

The complete genome sequence has been deposited in the DDBJ/EMBL/GenBank database under GenBank accession no. AP017652.
